# Snow droughts, deluge, and reservation systems interact to drive recreation access at Yosemite National Park

**DOI:** 10.1038/s41598-025-21022-5

**Published:** 2025-10-23

**Authors:** Jeffrey S. Jenkins, Adrienne M. Marshall, Sheri A. Shiflett, Rachel F. Mattos, Isaac T. Sanchez, Nicole D. Athearn

**Affiliations:** 1 Department of Management of Complex Systems, School of Engineering, University of California, Merced, 5200 Lake Rd, Merced, CA 95343 USA; 2 Hydrologic Science and Engineering Program, Colorado School of Mines, Golden, CO 80401 USA; 3 Division of Resources Management and Science, Yosemite National Park, 5083 Foresta Rd, El Portal, CA 95318 USA

**Keywords:** Snow deluge, Climate extreme, Snow water equivalent, Outdoor recreation, Use limits, Visitor use management, Hydrology, Climate change, Climate-change impacts

## Abstract

Changes in snow extremes can have important but as yet underexplored impacts on the 1.2-trillion dollar outdoor recreation industry, impacting essential ecosystem services. These hydroclimate conditions interact with reservation systems used by park managers to manage potential impacts to park resources and visitor experiences. Climate change extremes and associated hazards limit and enable access in different ways: snowpack from extreme wet years can prolong road closures at higher elevations, while extreme snow drought enables early season access. Few studies have attempted to assess and compare the influence of snow conditions and reservation systems among a population of visitors, as we do at Yosemite National Park. Roads were still closed into the 2023 peak season from a record extreme snow deluge, yet entry was unrestricted due to lack of a parkwide day use reservation system. This combination led to higher overall visitation levels but spatially constrained visitor mobility and resulted in more crowded conditions and traffic congestion. We assess the efficacy of the day use reservation system in limiting use by comparing differences from the annual mean and monthly volume and timing of visitors. Results show that annual use levels are influenced more by managed access than by climate extremes, and that results differ by overnight use types and traffic count locations with seasonal access limitations. These results demonstrate a novel aspect of the impacts of changing climate extremes with human use of natural resources and suggest that appropriately managed reservation systems are an essential tool for managing recreation resources in a changing climate.

**Supplementary Information:**

The online version contains supplementary material available at 10.1038/s41598-025-21022-5.

## Introduction

### Hydroclimate and water-based recreation

Changing snow conditions affect a broad suite of water-related ecosystem services spanning provision, regulation, and cultural services^1^. Impacts to water-related recreation can be large^1^, altering the socio-hydrological system^2^ in ways that are not yet fully understood^3^. Existing evidence suggests that hydroclimate changes impact recreational activities, potentially increasing visitation to parks in the U.S. and Canada^4–6^ in all but the warmest conditions^7,8^. Hydroclimatic changes can also alter the timing of visitation^9^, with particularly strong effects on both quantity and timing of visitation in extreme wet and dry years^10^b. Particularly high and low snow years, termed snow drought^11^ and snow deluge^12^ may be particularly impactful to outdoor recreation in the western U.S. when they interact with evolving recreation management systems, but these dynamics have not been fully explored.

Outdoor recreation and national park visits in mid to high latitudes are linked to seasonal variability, which differs by regional geographic and climate factors and their collective influence on tourist activities and the nature-based amenities that support them^13,14^. For mountain parks in the western United States, visitation typically ramps up in the spring and peaks in summer months, when temperatures are ideal for most recreationists and precipitation is limited. Heavy snowfall, icy roads, and resulting seasonal road closures limit vehicle access to mountain areas during the peak of winter, and snowpack can limit access to many trails and popular sites well into the spring^10,15^.

Visitors often consider recent climatic conditions when selecting a destination, formulating their plans on when and where to travel for different activities from various data inputs that influence their expectations, including official channels like park websites and marketing materials, as well as through social media^16,17^. Moreover, weather remains one of the most consumed topics in the media by winter recreationists, largely sourced through the Internet or mobile devices^18^. Tourists plan their trips around forecasts of local and regional weather, and often adjust their trip timing, trip length, or activities based on the weather^19^.

Previous studies have found that daily or monthly precipitation affects patterns of visitation, with visitation generally declining with more precipitation^8,20^. While precipitation reduces visitation while it is occurring, the resulting water increase directly or indirectly supports many outdoor recreation activities later in the season. Precipitation and snowpack may also affect the timing or prevalence of scenic resources in parks where visitation is correlated with seasonal water availability and runoff^21,22^. Quality waterfall viewing, for example, depends on a combination of snowpack and spring melt timing. Recreationists who enjoy swimming or walking near a river or lake generally prefer moderate streamflow and lake levels, with exceptionally low water levels often reducing access or enjoyment^23^.

Amidst changing hydroclimate-recreation interactions, managed access systems with advance reservation and time-based entry requirements have recently been implemented at national parks facing persistently high peak season demand and where there is potential for resources impairment^24^. These reservation systems likely interact with hydroclimate impacts on recreation in ways that are not yet well understood. Reservation systems are just one means for how recreational opportunities can be distributed. Recreational use may be rationed through different approaches, including reservation systems for different purposes (vehicle, front country, backcountry), lotteries (early access, public), booking windows, first come first served, or the use of pricing to reduce overall use and congestion to a desired level based on willingness to pay^25,26^. Park capacity allotment among different user groups (e.g., private bus tour groups, public day users, overnight backpacking lottery, educational group entry) on a given day, and rationing of total public recreation opportunities within each group, are a non-market exercise that can become a fraught political-legal process since demand cannot be curbed simply by increasing prices^27^ and the attempt to do so promotes inequity in public access.

The day use reservation system was first introduced to Yosemite in 2020, when a temporary online permit system was implemented in June following several months of full closure due to public health safety concerns. The vehicle-based system initially set visitation targets at 50% of the number of entries that were recorded in that month of the previous year (i.e., 2019)^28^. The COVID-era visitation targets were increased in 2021 to align with California’s county level risk tier system (e.g., 70%, 80%). In subsequent years (2022, 2024), the pilot “peak hours” day use reservation system was in effect daily during high-demand months (approx. mid-June to mid-September) to improve visitor experience by distributing rather than limiting visitation. Aspects of the reservation systems have changed from year to year, including flexibility of re-entry and arrival windows in response to the park’s monitoring of visitor re-entry patterns, target attainment, and other factors. The system doesn’t include overnight wilderness users, those staying overnight at park lodging, or those entering through public transit. The Visitor Access Management Plan (VAMP) was developed to reduce perennially overcrowded conditions and traffic congestion and to prevent resource impairment thresholds from being reached^29^. The VAMP was developed with comprehensive scientific and managerial input and went through an extensive public comment process in 2024, but has not yet been implemented as of the date of this publication.

**Fig. 1:**
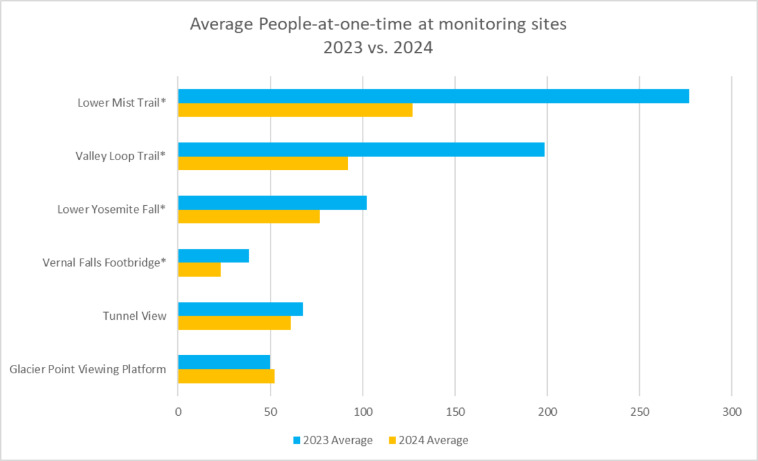
People-at-one-time visitor monitoring counts for 2023 and 2024 at popular viewing areas and trail segments. Asterisks denote viewing locations that are directly dependent on water features.

**Fig. 2:**
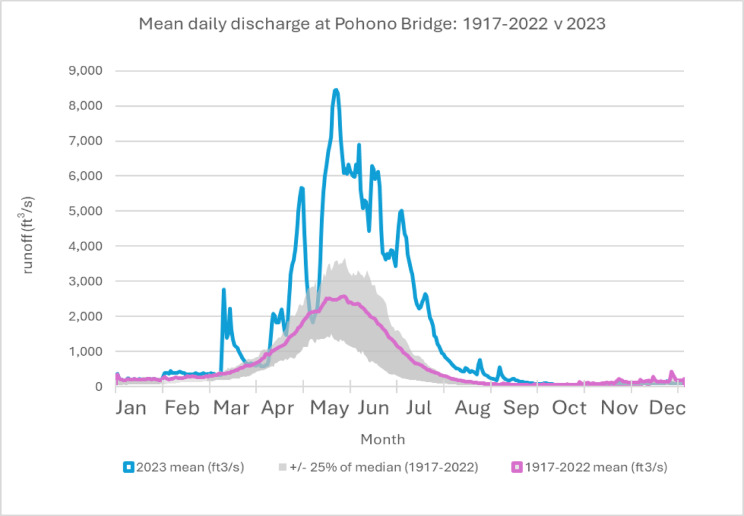
Mean daily discharge (ft^3^/s) of Merced River at Pohono Bridge, 1917–2022 versus 2023, gauge no. 11266500.

Visitor use data from 2023 and 2024 suggests that recreation behavior was amplified by demand for water features associated with extreme wet conditions in 2023. One aspect of resources monitoring for the VAMP included observing the number of visitors present at popular viewpoints and trail segments at different times of the day during peak season at the park. Notably, locations along the Merced River and waterfall viewing areas where viewing was directly dependent on water (i.e., Vernal Falls footbridge, Lower Mist Trail, Valley Loop Trail, and Lower Yosemite Falls) had substantially higher use during 2023 than in 2024, especially when compared with locations beyond the Valley without direct access to water features (i.e., Glacier Point and Tunnel View) that didn’t see a notable difference in use between the years (Fig. 1). While park access was limited in winter and early spring due to flooding and associated hazards that damaged infrastructure and led to park closures, higher-than-average run-off sustained water features throughout the peak summer season (Fig. 2)^30^

### Climatic influences on visitor access

Snow surveyors in California described the 2023 April 1 snow water equivalent (SWE) as the largest state-wide average since 1921 per modeled data, and potentially further back, considering challenges in comparisons given changes in the observational networks^31^. The largest values of April 1 SWE were recorded in the central and southern Sierra Nevada with subsequent flooding impacts to foothill and valley communities in spring^12^. Winter storms that year led to intermittent park closures throughout the Spring as infrastructure was damaged and roads became hazards, culminating in May with a 200-ft long fissure across a primary park road (Big Oak Flat Road, the continuation of Highway 120 from the western park entrance) that cut off access to Yosemite Valley. That summer was the first peak season to not have a day use reservation system in place since being introduced during COVID, but access to the high country was limited by snowpack, resulting in the latest opening of Tioga Pass Rd. to date: July 22. The combination of no park-wide day use reservation system in place and road closures preventing access to higher elevation corridors that visitors typically expect to be open led to higher overall levels of use but spatially constrained mobility, resulting in more crowded conditions and increased traffic congestion in Yosemite Valley.

**Fig. 3:**
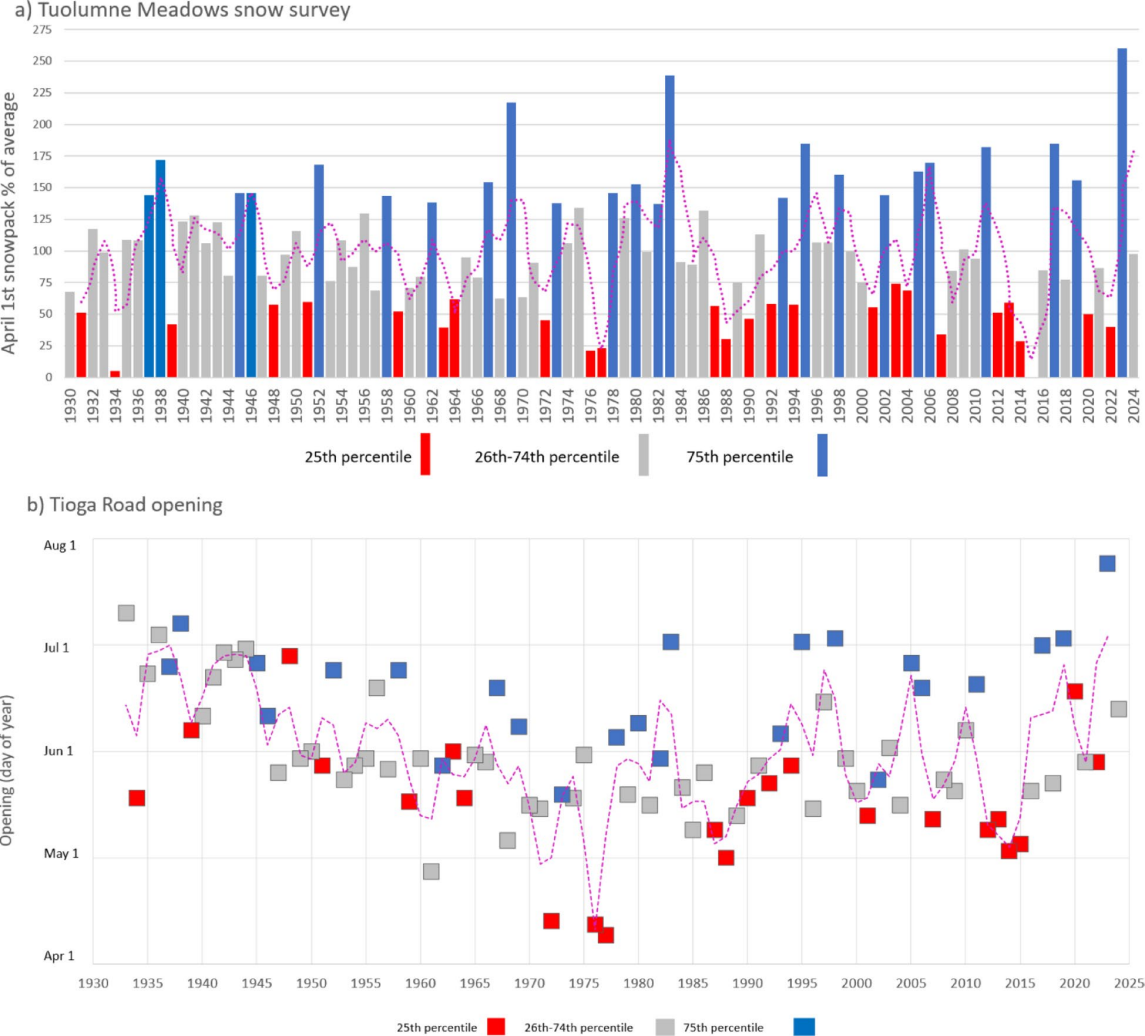
(**a**) Percent of average April 1 SWE for Tuolumne Meadows snow course, and (**b**) Tioga Road opening day of year by April 1 SWE. Snow drought years (25th percentile is 62% of average) are shown with red, high snowpack years (75th percentile is 135% of average) are shown with blue, and near average snowpack years (62–135%) are shown with gray. Dashed lines show a running average of 2 years, chosen to account for antecedent conditions of the previous season and visualize variability over time.

The year 2023 stands in contrast to the long-term drought conditions facing the region, including snow drought when anomalously low snowpack results in water resources scarcity and has consequences for ecosystem function^11,32^. While snowpack has historically displayed high annual variability in the Sierra Nevada, where Yosemite NP is located (Fig. 3a), a shift toward megadrought across the broader southwestern United States since 2000 has resulted in lower and downward trending snowpack^33,34^. Visitor and manager expectations for the timing of accessibility to high elevation areas are adapting to anticipate lower snowpack, planning tentatively for earlier road openings that provide access to higher elevation destinations despite historically uncertain snowpack and trail conditions^15^. Such is the case with Tioga Rd. in Yosemite NP, where snow drought is associated with earlier plowing and road opening, while wet conditions lead to later openings (Fig. 3b). These seasonal differences have important implications for managing the flow of visitors and resource protection as winter and spring snowpack recedes, snow drought becomes more frequent, and the annual timing of peak snowpack becomes more variable^35,36^.

Despite a downward trend in snowpack, and fewer expected smaller snowfall events, extreme snowfall events are projected to decline less and could even increase in cold regions^37,38^. While in 2023 there was no singular event like that of the 1997 (when discharge exceeded 10,000 ft3/s due to warm rainfall that contributed to rapid snowpack melting), there were multiple extreme flooding events early in the season followed by prolonged high levels of runoff driven by snowpack melting into the late summer months^39^. Atmospheric rivers, where water vapor is transported through long, narrow regions in the atmosphere, contributed to the record precipitation and snowfall that resulted in immediate impacts, including pervasive flooding^40^. Very large snow accumulations can occur in years with a high frequency of atmospheric rivers in the western United States, though most snowfall studies have focused on the event scale rather than overall seasonal accumulation^41,42^. Individual extreme snowfall events account for the majority of interannual variability in snowpack, particularly in California as compared to the rest of the West^43,44^.

In this study we assess the effect between years of snow drought and deluge conditions on timing and volume of peak visitors and compare these during the historically wet 2023 season, which had no entry limits, and with other recent years (2020, 2021, 2022, 2024) that had reservations in place and happened to be all drier years. To assess the efficacy of the reservation systems in limiting overall park use, we hypothesize that there is a greater difference from average annual visitation for an extreme wet year without reservations (2023) and recent years with reservations in place that happen to be moderately dry than for years of extreme wet and extreme drought conditions. We assess differences based on annual aggregation of monthly visitor data in three contexts: (1) at the park-wide level to assess overall effect; (2) by overnight use types (lodging, RV, camping, backcountry) that range in visitor exposure to environmental conditions; and (3) by vehicle access, comparing year round entry gates with vehicle counts at high elevation seasonal locations, where access is dependent on snowpack.

## Materials and methods

### Study area

**Fig. 4:**
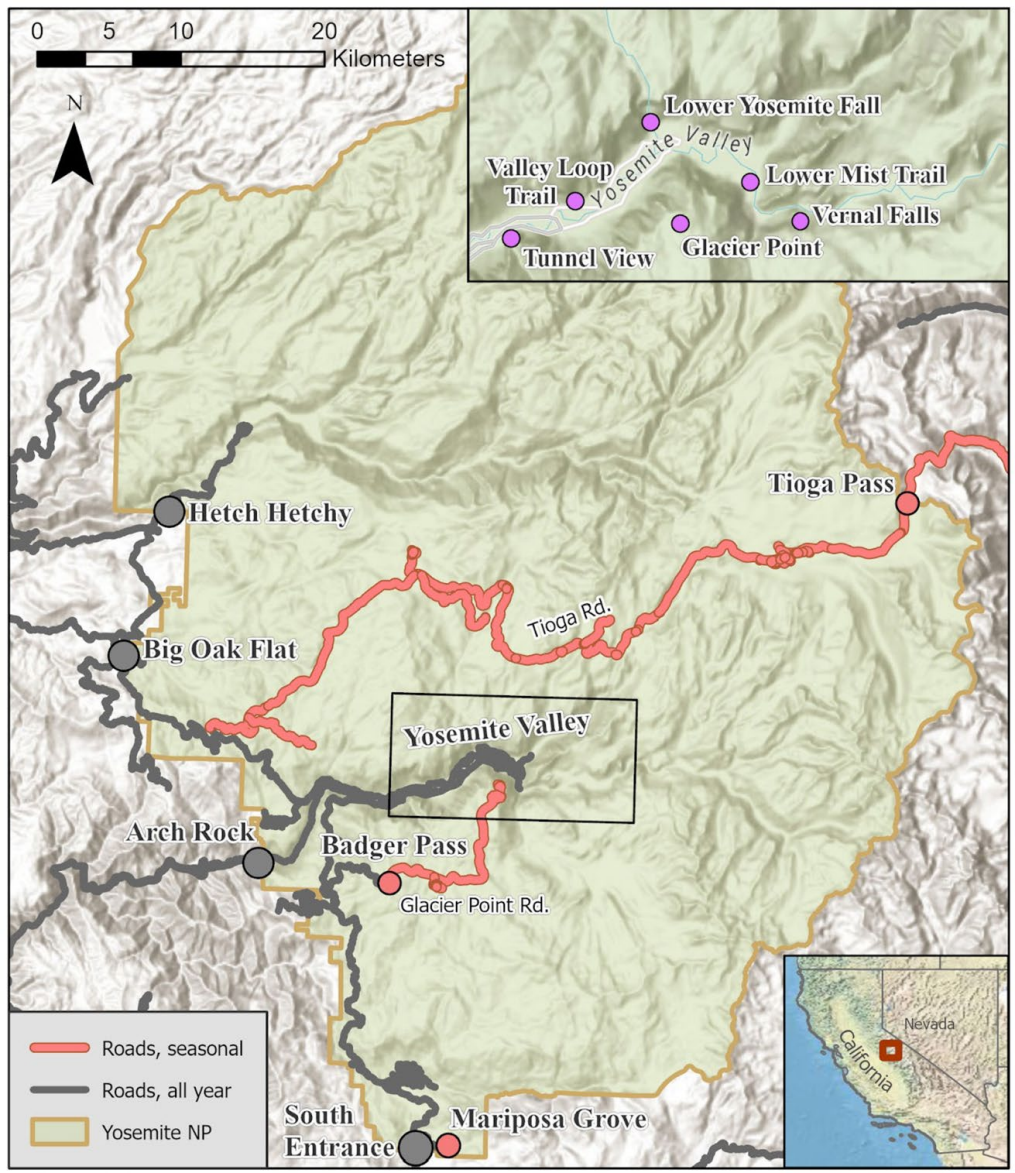
Yosemite NP with year-round and seasonal roads and locations. The upper inset shows locations of people-at-one-time monitoring in Yosemite Valley. Map by author, data source: Esri, World Hill Shade [basemap], World Topographic Map (accessed 2025). ArcGIS Pro: Version 3.3.

Yosemite National Park (NP) receives millions of visitors each year and constitutes an important node in recreation ecosystems^3^ and the socio-hydrology of California. Yosemite NP was an early innovator of managed access, beginning with the overnight wilderness permit system that was made compulsory in 1972, with limits and enforcement introduced in subsequent years^45^. The Park implemented its General Management Plan in 1985 in recognition of the need to reduce traffic and control the intensity of visitor use in the Yosemite Valley, Mariposa Grove, and other hotspots through planned removal and redesign of vehicular infrastructure and visitor facilities (Fig. 4). Visitation continued to grow over the next decade; by 1987, the annual park-wide use at Yosemite surpassed 3-million visitors, and by 1996 there were more than 4-million visitors to the Park^46^.

Additionally, the Merced River Plan, released in 2014, did not require river managers to set park-wide capacities, rather the park developed plans for specific locations, including to manage the river corridor pursuant to the Wild and Scenic Rivers Act. To do this the Park Service proposed a user capacity program establishing procedures for monitoring conditions of park resources and visitor experiences, where visitation limits were based on facility capacities, instead of capping public access to Yosemite^47^. In 2016 use surpassed 5-million visitors, owing in part to the centennial of the National Park Service^48^. Meanwhile, prolonged multi-year drought conditions expanded seasonal access to higher elevation locations and helped to drive record visitation to the park, despite poor air quality from fires burning that year^40,49^.

### Data

Historic visitation data are available for units of the National Park System through the Integrated Resource Management Applications (IRMA) Visitor Use Statistics portal^48^. For Yosemite NP we utilized IRMA data available for (1) monthly total entries into the park (1980–2024), (2) park-wide overnight use by lodging type (1980–2023), and (3) vehicle counts (1985–2024). Overnight lodging types include concessionaire lodging, RV campers, front country tent campers, and backcountry wilderness camping. Each category represents a monthly park-wide total for that type of overnight use. Some overnight activities expose users more to environmental conditions than others, resulting in visitation types having variable sensitivity to climatic conditions. Monthly vehicle counts are taken from park entry points that are typically accessible year-round, including Arch Rock, Big Oak Flat, Hetch Hetchy, and South Entrance, as well as entries and parking areas that are seasonally accessible due to snow constraints, including Badger Pass, Mariposa Grove, and Tioga Pass. Year-round access points may become temporarily closed due to hazards within the park (e.g., active fire or flood) or impacts on the roads themselves (e.g., geologic events like rockslides or a road crack).

**Fig. 5:**
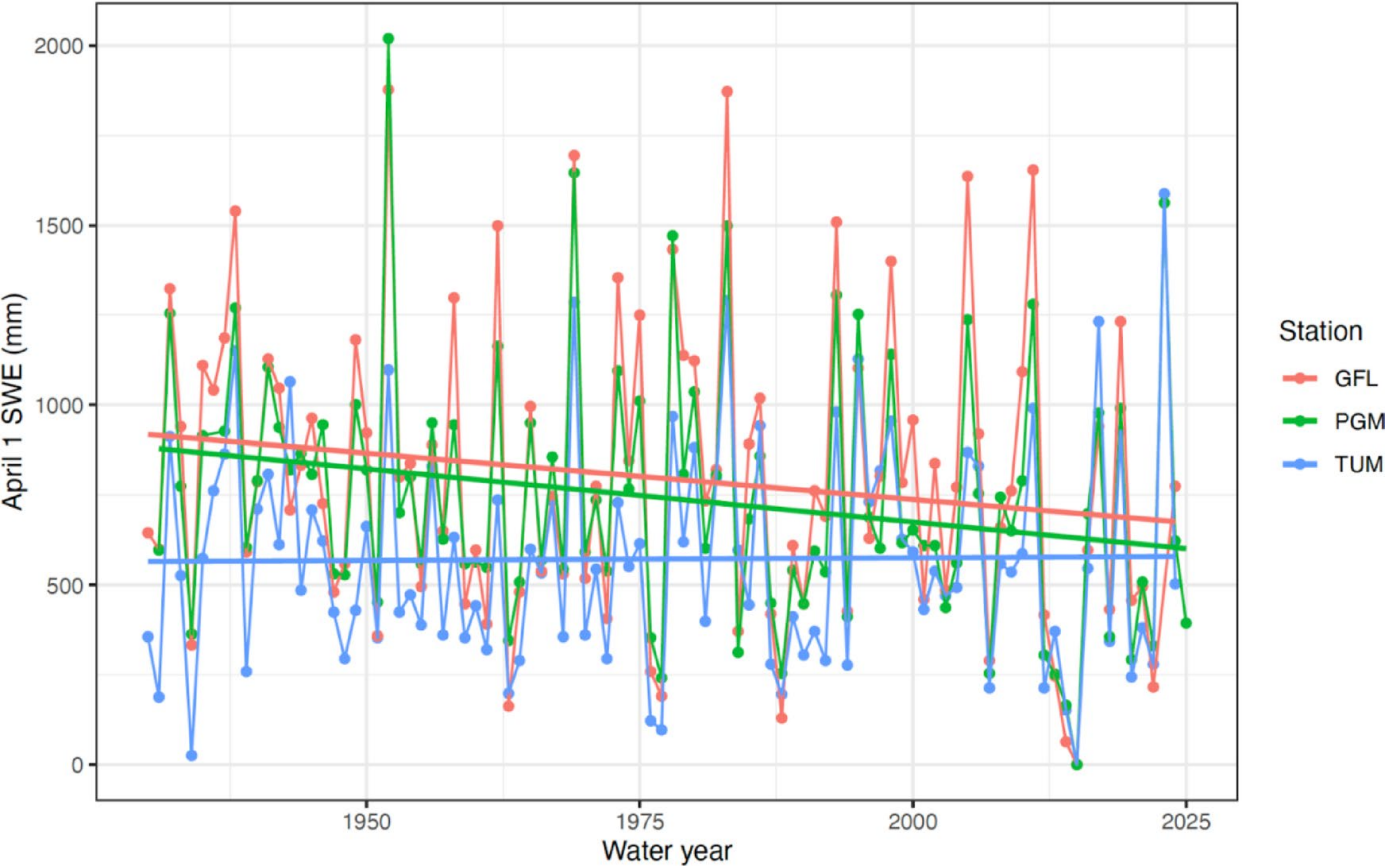
April 1 SWE data from three snow courses closest to major visitor locations in Yosemite. PGM = Peregoy Meadows, TUM = Tuolumne Meadows, GFL = Gin Flat. Lines indicate linear regression slopes (conditional mean) for each station, with April 1 SWE estimated as a linear function of water year.

Data were utilized from three snow courses in Yosemite NP with long periods of record (1980–2024) for April 1 SWE, including for Gin Flat (GFL) near Tuolumne Grove, Peregoy Meadows (PGM) near Badger Pass, and Tuolumne Meadows (TUM). The April 1 SWE is a standard metric for water supply forecasts and coincides often with maximum SWE across mountains of the western United States^50^. We use the measurement as a sentinel for snowpack across the park, given use in previous studies^15,51^ and that the April 1 snow survey is used by Yosemite NP managers as a key indicator for conditions and visitor access. Data are collected as part of a system of snow surveys conducted on the first of the month, typically four to five times in the winter, and records of snow course data are kept accessible through the California Department of Water Resources^52^**.** Snow course locations are geographically representative of the higher use transportation corridors and visitation zones of the park: GFL as part of the northwestern zone, PGM as part of the southern zone and closest to Yosemite Valley, and TUM for the north-central high country. The percentage of average and SWE in inches between these three sites were compared using correlation analysis to determine if the trends over time are comparable between sites (Fig. 5). There is a high correlation (0.84–0.95) among the different snow courses over time, indicating that elevation differences between sites have a minimal effect on interannual anomalies, and that the data from one site may be representative of relative change over time across all. Given the high correlations, TUM is used as a sentinel of park-wide SWE data, and we use it throughout our analyses for consistency. Furthermore, the annual spring snow course sampling efforts by wilderness rangers at TUM are often communicated to the public through the Park’s website and via social media to help inform visitors about the most likely spring conditions throughout the Park, and visitors use this information in conjunction with snow plowing reports to anticipate the opening timing of Tioga Road, which provides access to the high country, and as an indicator to assess potential conditions throughout much of the Park.

### Methods

Our analysis assesses the influence of extreme snow conditions and managed access (i.e., day use reservations) on visitor volume and timing for (1) park-wide use, (2) type of overnight lodging, and (3) traffic counts at entry gates and seasonal roads. Across these datasets, we assign categories to each year from 1980 to 2023 based on April 1 SWE conditions, snow drought or deluge, or by managed access status, between the snow deluge year of 2023 with no entry limits in place and other recent years with reservations—2020, 2021, 2022, 2024, which range from near normal climate to moderate drought conditions. Snow drought and deluge are derived by grouping conditions into three categories of SWE, which allows for greater comparison of extreme drought and wet conditions, as has been done effectively in other studies^10,15,53^. We assign a category for each year based on percent of average April 1 SWE at TUM: < 50% of average for extreme dry conditions, 50%–150% of average for average-moderate conditions, and > 150% of average for extreme wet conditions. We used these categorical definitions, rather than SWE as a continuous variable based on our expectation that extreme SWE years would be impactful but that visitation would not likely otherwise be a linear function of April 1 SWE. Visitation data from extreme wet and extreme drought years are each aggregated and averaged by month, along with the average monthly visitation under managed access status and monthly values from 2023. Note that as 2023 is an extreme wet year, it is also grouped within the category of extreme wet years, which provides for a more standardized comparative approach given that the average of all years in the data set remain unchanged regardless of how categories are binned by naming convention.

We first find the annual difference from the mean for each category, which allows us to compare relative impact between categories for park-wide visitation, overnight lodging types, and vehicle traffic count locations, year-round and seasonal. Results are then plotted by month for each IRMA dataset and traffic location or type of overnight use to visualize differences in timing and volume of visitors between extreme climate conditions and managed access status. We use months as a unit of analysis as this is the highest temporal resolution available in the reported visitation data from IRMA. Months are uniquely suited as a time scale of analysis for visitation data, particularly for mountain parks that experience differences in visitation levels by season as snowpack restricts access to roads and facilities, and are thus useful to compare the timing of visitor access from year-to-year^54^. Potential visitors and park managers themselves hold expectations about what locations can be accessed and how many people might be visiting for a typical month, and these expectations are widely exchanged and reified through personal experience and available information^16,33^.

We estimate three regression models to assess how annual visitation is impacted by snowpack drought and deluge and reservation type. Regression models are estimated separately for: (1) overall visitors from 1980 to 2024, (2) type of overnight lodging from 1980 to 2023, and (3) traffic counts at year-round and seasonal park entryways from 1985 to 2024. Each response variable was transformed to a z-score to facilitate comparisons across the visitation domains following^55^. Predictor variables include snow category, policy category and their interaction (Policy category x Snow category), allowing us to test whether the effect of policy varies under different snow conditions.

$$Y={\upbeta }_{0}+{\upbeta }_{1}C+{\upbeta }_{2}P+{\upbeta }_{3}CP+\upepsilon$$

*Y* = Annual z-score for each visitation category; *C* = Snow category (snow deluge, snow drought); *P* = Policy category (Reservations, No Limits); ∈  = Error term.

For each regression, standardized slopes (β) were calculated using ordinary least squares within our model (OLS). Parametric 95% confidence intervals (CI) were constructed; the slopes for each term were evaluated to assess whether confidence intervals excluded zero and for statistical significance when *p* < 0.05. We also evaluated the R^2^ for each model. CI significance metrics are useful in nature-based recreation as they more transparently convey the direction, magnitude and uncertainty associated with policy and climate effects on visitation outcomes^56,57^. All analyses were conducted in R^58^.

## Results and discussion

**Fig. 6:**
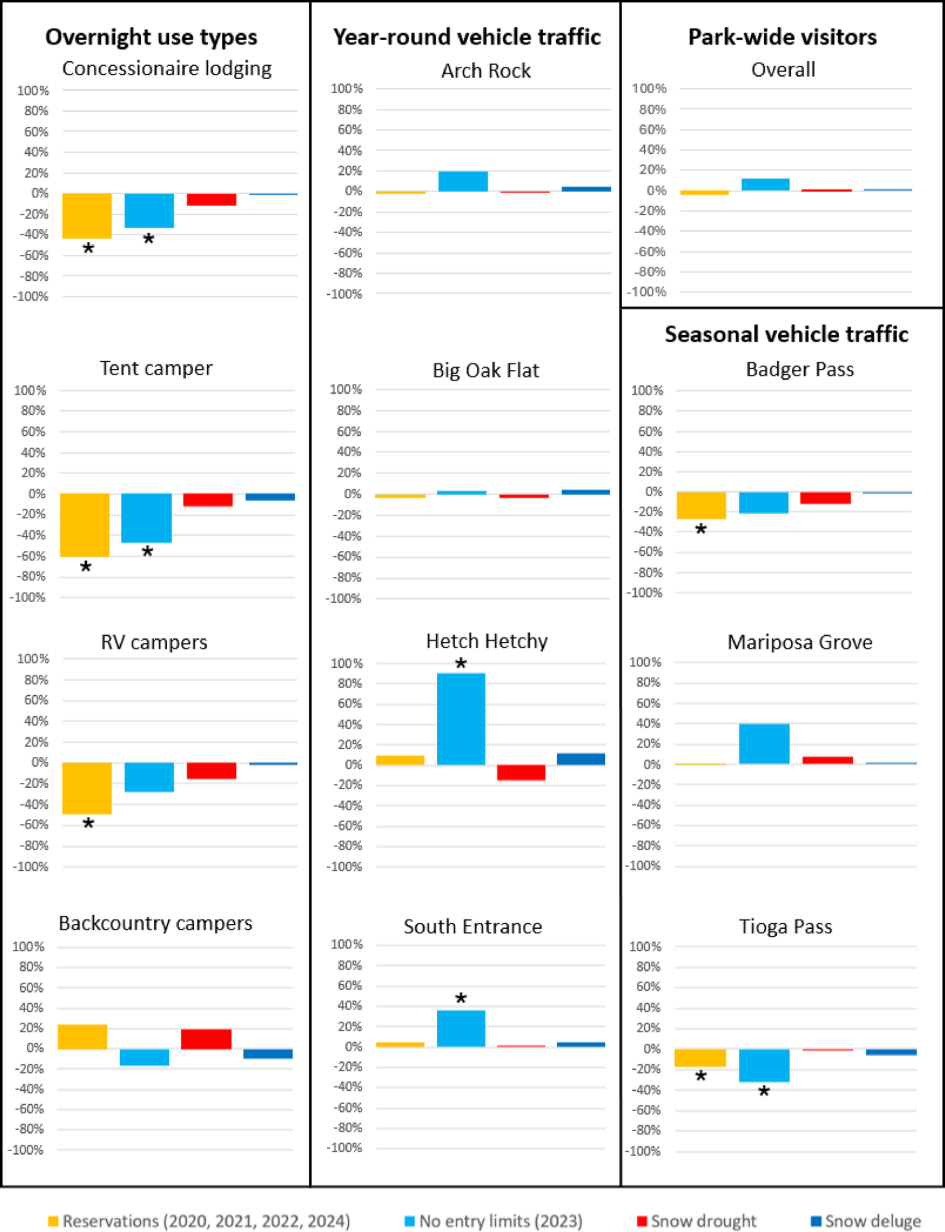
Percent difference from annual mean of visitation (1980–2024) for overall park-wide visitation, types of overnight use, and vehicle traffic counts for year-round entrances and seasonally accessible locations by recent managed access status and by climate conditions. Asterisks indicate statistical significance (CI 95%).

Regression analyses for the three separate models: park-wide use, type of overnight lodging and vehicle traffic yielded overall insignificant effects (*p* > 0.05), although individual terms were significant for predicting overnight lodging type and vehicle traffic (Table 1, Fig. 6). This discrepancy implies that our model has limited predictive power overall and some included predictor variables may be orthogonal to the response but does not prevent interpretation of the effect directions of individual variables. Interaction terms between reservation policy and snowpack category: i.e., Reservation Policy x Snow Deluge Conditions & Reservation Policy x Snow Drought Conditions; were nonsignificant across all domains (*p* > 0.05) and are therefore not displayed in the final tables.

**Table 1:** Regression results: model fit statistics and significant effects.

Type	R^2^	Adj. R^2^	F-stat	df_1_	df_2_	*p*-value	Formula	
Park-wide Visits	0.038	− 0.056	0.407	4	41	0.802	F(4, 41) = 0.407, *p* = 0.802	
Overnight Lodging	0.042	0.019	1.858	4	171	0.12	F(4, 171) = 1.858, *p* = 0.12	
Vehicle Traffic	0.003	− 0.012	0.2	4	275	0.938	F(4, 275) = 0.200, *p* = 0.938	

Type	Group	Policy Type	β (std)	β (unstd)	Lower CI	Upper CI	*p*-value	CI Significance
Overnight Lodging	Concessionaire	Reservations	− 0.65664	− 1.379	− 1.879	− 0.88	0	*
	Concessionaire	No Reservations 2023	− 0.30959	− 1.099	− 1.93	− 0.269	0.011	*
	RV Campers	Reservations	− 0.47603	− 0.522	− 0.84	− 0.203	0.002	*
	Tent Campers	Reservations	− 0.65119	− 1.235	− 1.689	− 0.781	0	*
	Tent Campers	No Reservations 2023	− 0.30835	− 0.989	− 1.745	− 0.233	0.012	*
Vehicle Traffic	Badger Pass	Reservations	− 0.41502	− 0.518	− 0.92	− 0.115	0.013	*
	Hetch Hetchy	No Reservations 2023	0.411933	0.154	0.037	0.272	0.011	*
	South Entrance	No Reservations 2023	0.404312	1.168	0.262	2.074	0.013	*
	Tioga Pass	Reservations	− 0.47424	− 0.315	− 0.503	− 0.128	0.002	*
	Tioga Pass	No Reservations 2023	− 0.41326	− 0.528	− 0.879	− 0.177	0.004	*

R^2^, Adj. R^2^, F, df_1_, df_2_, and Model p represent model-level statistics. β (std) = standardized beta, β (unstd) = unstandardized estimate. CI Sig. =  ‘*’ when 95% CI excludes zero. All other regression model outputs, including non-significant results are in supplementary Table 1.

Across categories annual use levels are influenced more by managed access status than by climate extreme, and results differ in highly contextual ways by type of overnight use and by traffic count locations, some of which include seasonal access limitations due to snowpack.

Table 1 Summary of standardized linear regression results for Yosemite National Park (YNP) model statistics and key coefficients from the three visitation domains. Standardized beta coefficients suggest positive directional influence (+) and negative directional influence (−) under conditions and policy regimes.

**Fig. 7:**
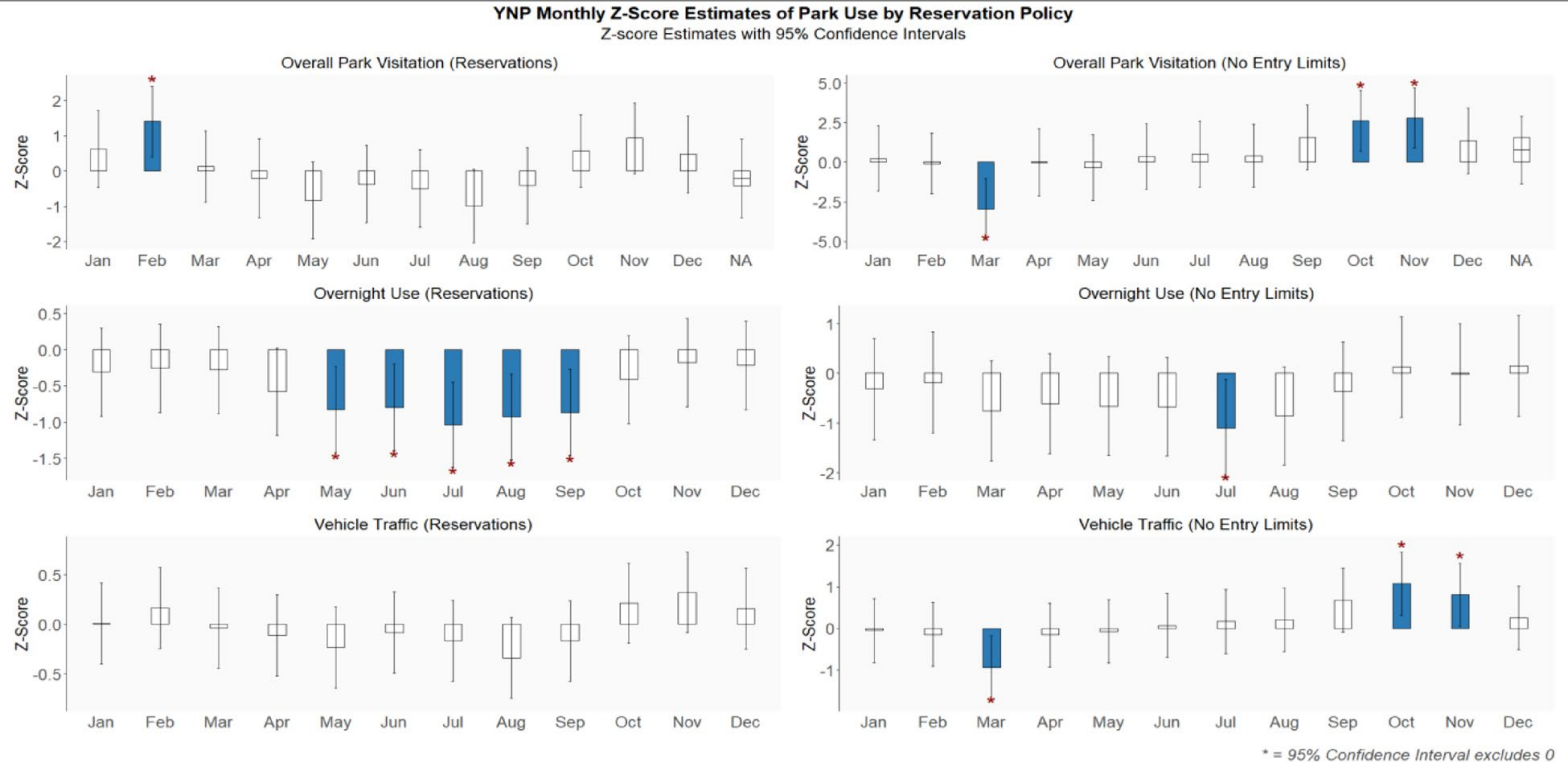
Yosemite National Park monthly visits by reservation policy. Reservations (2020, 2021, 2022 & 2024) and no entry limits (2023).

Park-wide visitation showed a statistically significant increase in February during reservation years (2020–2022, 2024), while overnight use during the same reservation regime showed decreases in overnight lodging. No entry limits (2023), a snow deluge year, showed Park-wide visitation down for the month of March, but positive significance in October and November. Vehicle traffic, mirrored that same significance, and overnight use showed significant trend during summer months under reservations and significance in July for no entry limits (Fig. 7). This suggests that overall, the reservation system was effective in accomplishing the intended goal of reducing the number of visitors during peak summer months, thereby mitigating crowded conditions and long traffic delays, and that despite visitors displacing to different days and seasons, the result of the peak season day use reservation system being in place was less overall use throughout the year.

In the overnight lodging analysis, backcountry camping was the only type of overnight use that exhibited marginal positive results under reservations; whereas all others: Concessionaire, Tent and RV campers were negatively and statistically affected under reservations and more so, Concessionaire and tent campers were also statistically significant under no entry limits. Overall park visitation was affected during reservation years, all of which had moderately dry conditions, and extreme drought conditions. Higher elevation destinations in Yosemite are more accessible, earlier during drought years, but importantly obtaining an overnight wilderness permit allows visitors to enter the park without a day use reservation (as does having overnight lodging or camping reservations), and since 2020 Yosemite NP has issued a record amount of wilderness permits, owing to this combination of drought and rationing system. Conversely, no entry limits (2023) showed a negative trend, indicating that increased snow severely limited early season backcountry use. For other overnight lodging types (tent camping, RVs, concessionaire lodging), categories of use limit years and no entry limits in 2023 showed more substantial negative change than extreme snow years.

Analysis of vehicle traffic counts revealed notable entrance specific effects. Big Oak Flat displayed statistically significant observations across all four conditions of—reservations, no entry limits, extreme drought and extreme wet; with decreased levels of access under extreme drought conditions and years when day use reservations have been in place, and increased use under extreme wet and no entry limits (2023). Big Oak Flat entrance has faced closures and reductions in access during extreme dry years due to fire, during extreme wet years due to debris flows from flooding in post-fire scars, and during extreme wet years less people access Big Oak Flat when Tioga Pass is closed. Other entrances, Hetch Hetchy and South Entrance showed statistically significant positive increases as well as the seasonally accessible Mariposa Grove showed positive increase under the no entry limits (2023) condition due to visitors being rerouted from Big Oak Flat entrance closure during spring from the 200-ft fissure in the road and displaced from Tioga Road due to its closure into late summer. In contrast, Badger Pass and Tioga Pass showed statistically significant downward shifts in traffic under no entry limits in 2023, likely due to closures or access limitations from extreme wet conditions in 2023. Collectively, the data indicate that both policy and climate conditions shape overnight stays, visitor access and routes taken into the park.

**Fig. 8:**
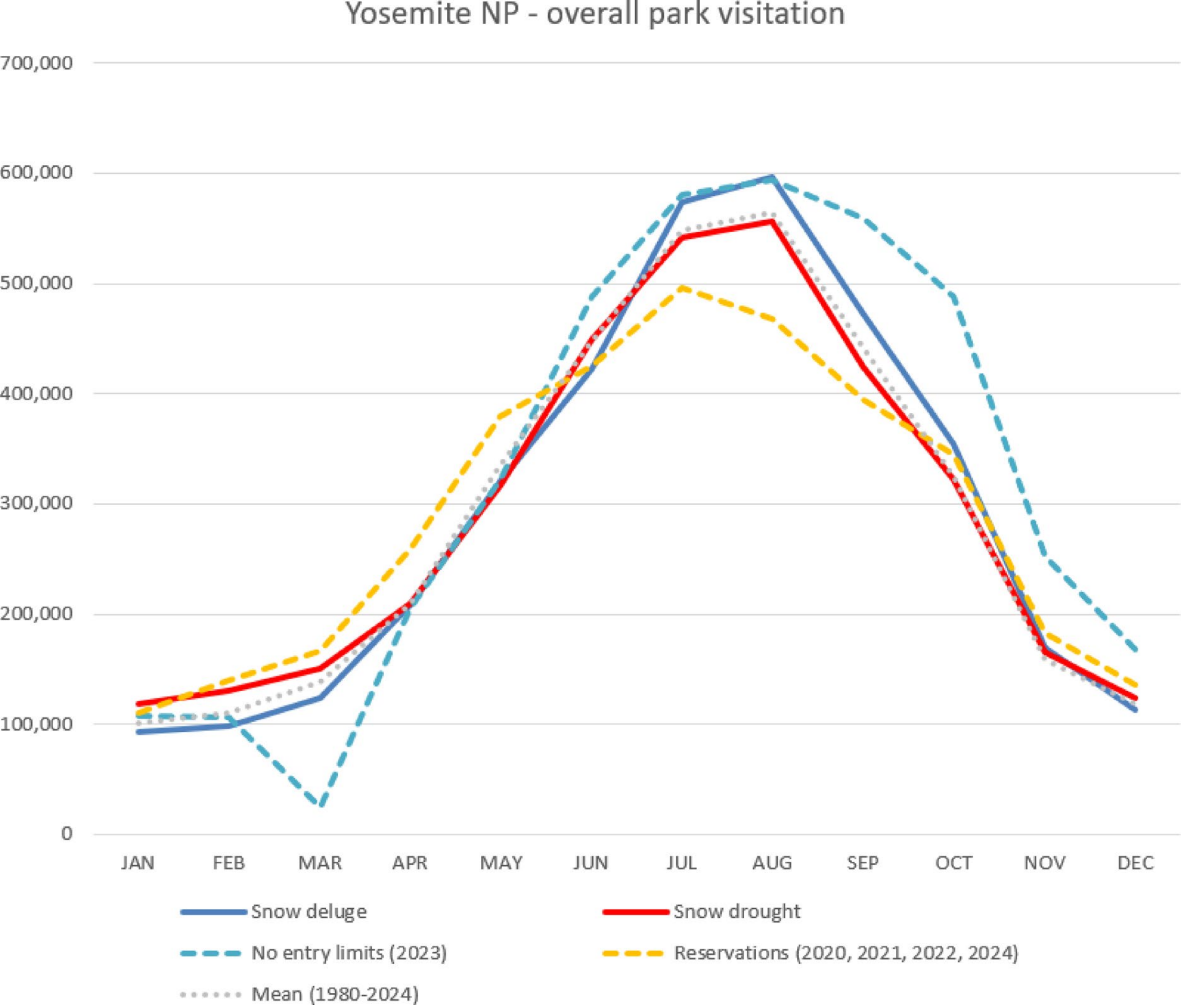
Average monthly park-wide visitation to Yosemite NP by climate conditions and managed access status.

The number of total annual visitors to the park during different climatic and management regimes varies minimally from average annual use, though 2023 saw + 11.8% more users than on average (Figs. 6, 7, 8). However, important differences in the timing of seasonal visitation exist between these different categories. Extreme wet conditions including flooding and hazards led to the Park’s closure in mid-January, late February, and the first-half of March, which displaced much of the would-be use during winter and resulted in heavy damage to infrastructure and buildings. In May campgrounds in the Yosemite Valley had to be evacuated and sites closed due to high levels of flooding from spring snowmelt, and the Big Oak Flat Road developed a large fissure and failed, leaving no access to most of the Park from Highway 120 to the west from May 2 to June 9. But the wet conditions earlier in the season and no entry limits led to high visitation throughout the peak summer months and remainder of the year, a displacement effect more pronounced than during other extreme wet years. Years where the reservation systems are in place during the peak months show less overall use in summer due to those limits but more use in late winter and higher levels of late-winter and spring use as visitors displace to a different season. In these cases, earlier seasonal access was facilitated by drier conditions during spring. Extreme drought years show slightly more use in the winter and spring afforded by less snowpack, but on average less use throughout the remainder of the season due to wildfires and associated closures.

**Fig. 9:**
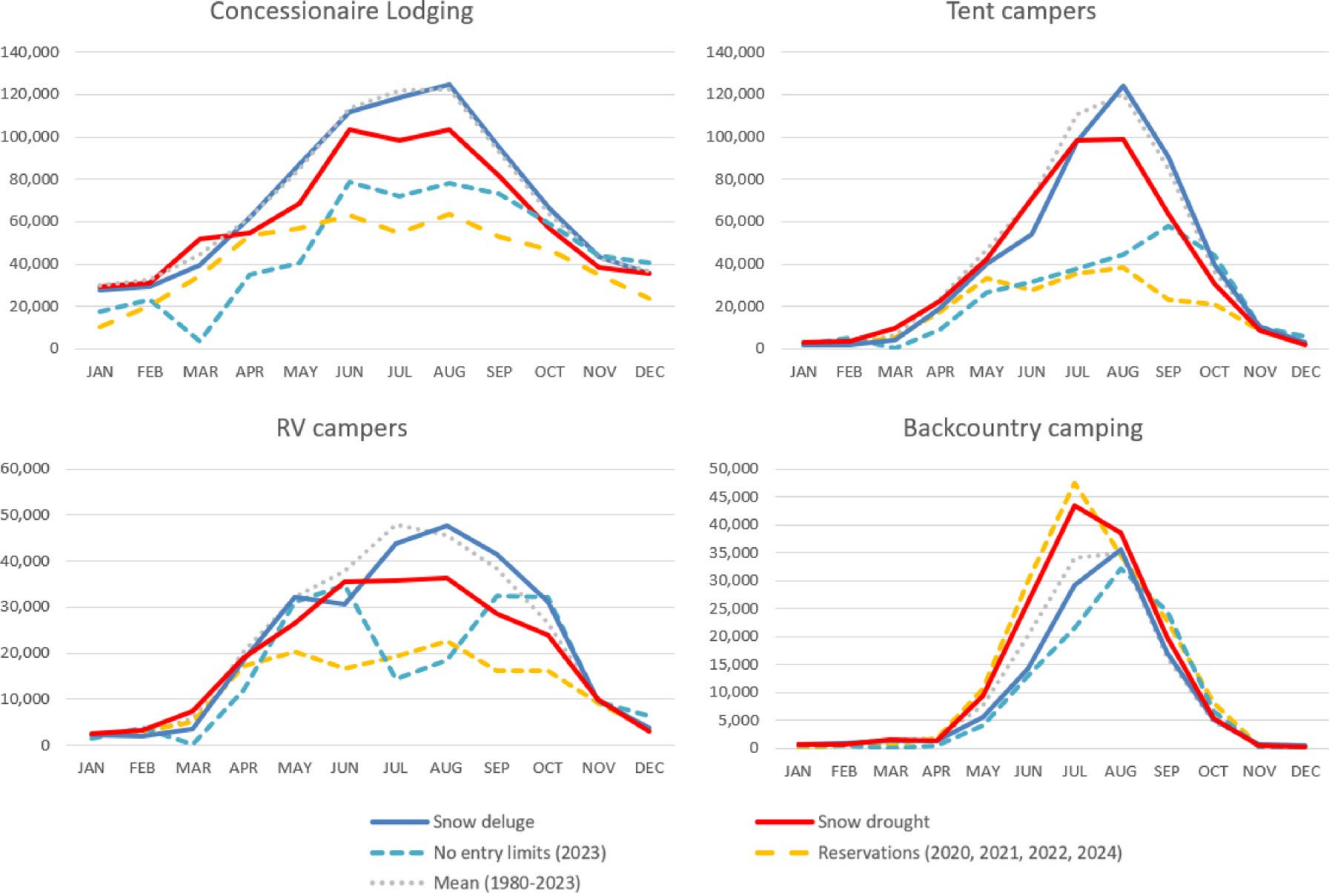
Average monthly overnight use at Yosemite NP (1980–2023) by climate conditions and managed access status for concessionaire lodging, tent campers, RV campers, and backcountry camping.

Overall, for concessionaire lodging and tent campers, management regimes of reservation system limits and winter storm closures during snow deluge years like 2023 have a greater effect on visitation than climate conditions, including snow drought or previous extreme wet condition years (Fig. 9). Concessionaire lodging has − 43.0% of annual visitors during reservation years compared with − 33.2% of visitors in 2023. Tent campers have − 60.9% of annual visitors during reservation years compared with − 46.8% of visitors in 2023. This may be due to several factors including that use is limited during peak season reservation periods, and that many of the lodging areas and campgrounds are exposed to hazards and cannot operate during winter storm closures.

Importantly, the campground areas accessed from Tioga Road weren’t open or accessible until Tioga Road opened for the season after snow plowing, which in 2023 was the latest opening ever recorded. More use occurs during snow deluge years when summer visitation is not as limited by the effects of wildfire (e.g., smoke, road closures) like in snow drought years. However, visitation differences between these hydroclimate categories are minimal, which reflects saturated demand for overnight use within the park: lodging reservation is often booked a year in advance and campground reservations 5 months ahead of time. Conversely, RV campers and backcountry campers have different flexibilities and limitations which constrain or enable access. Overnight RVs were − 49.3% of average annual use during the day use reservation system in place as vehicle entry limits were imposed. RV overnight use in the park was drastically less in 2023 peak season than in any other year (− 27.9% across the year) even though there were no entry limits, however 2023 was the latest day on record for Tioga Pass Rd opening (July 22nd).

Whereas other extreme wet years have high overnight RV use throughout the summer as visitors connect parks as part of a road trip, the extremely late opening of Tioga Pass Rd resulted in far fewer RVs being able to cross into the park from the eastern side and instead forgo their visit. Backcountry overnight use is always far less, far later with extreme wet conditions resulting in high snowpack thereby limiting access to the high country. Extreme drought and drier years allow for earlier access and more use throughout summer with the highest number of overnight wilderness users during park-wide reservation years (+ 23.0% more than annual average). Backcountry camping requires an overnight wilderness permit, which is allocated through a different permit system than the park’s day use reservation system and not subject to the park entry limits. As such, the volume and timing of wilderness use is relatively indifferent to the management regime—indeed high levels of use and associated crowding, or inability to get a day use reservation might drive some potential front country users to a wilderness permit instead.

**Fig. 10:**
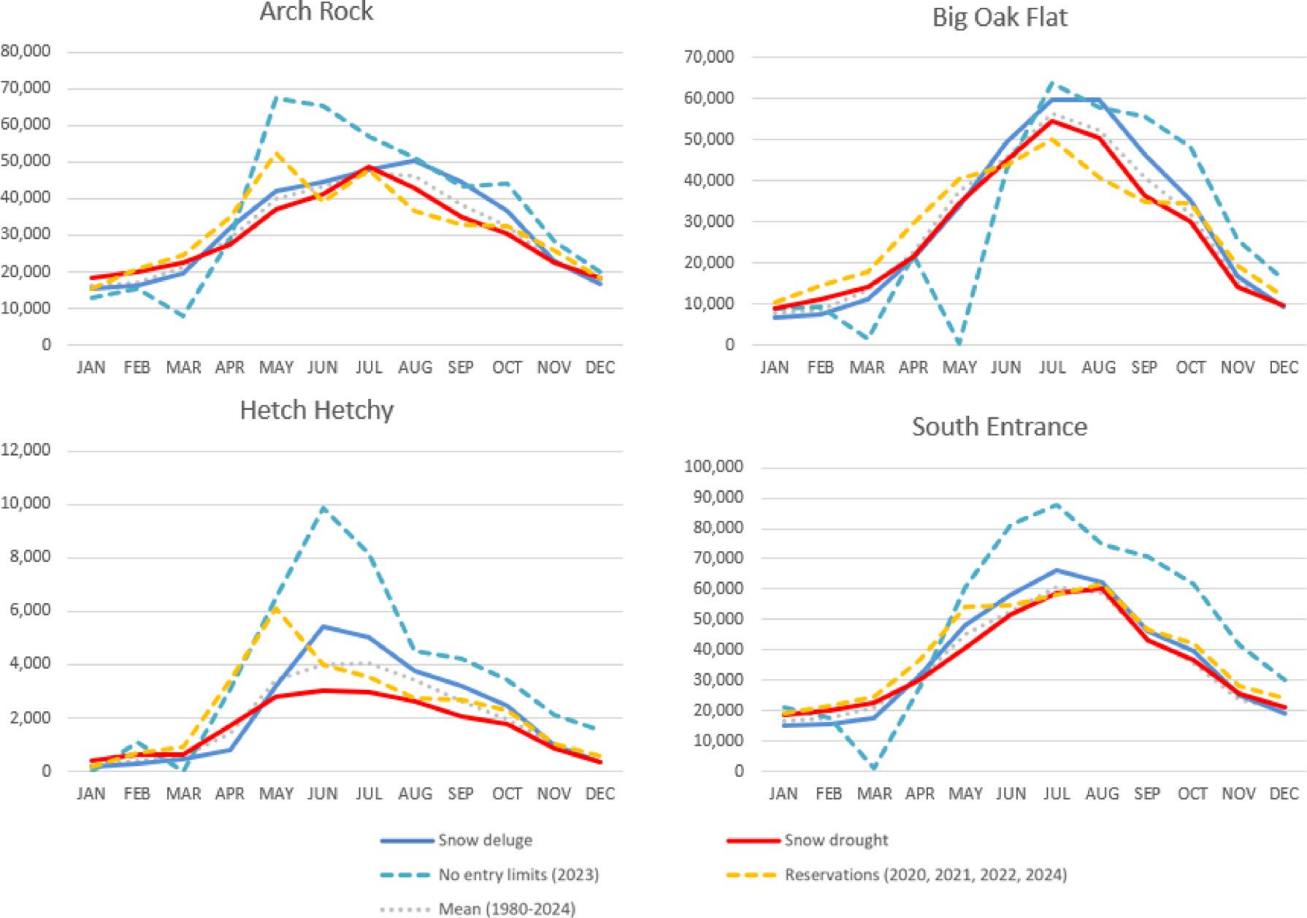
Average monthly vehicle counts at Yosemite NP (1985–2024) by climate conditions and managed access status for year-round entrance stations.

**Fig. 11:**
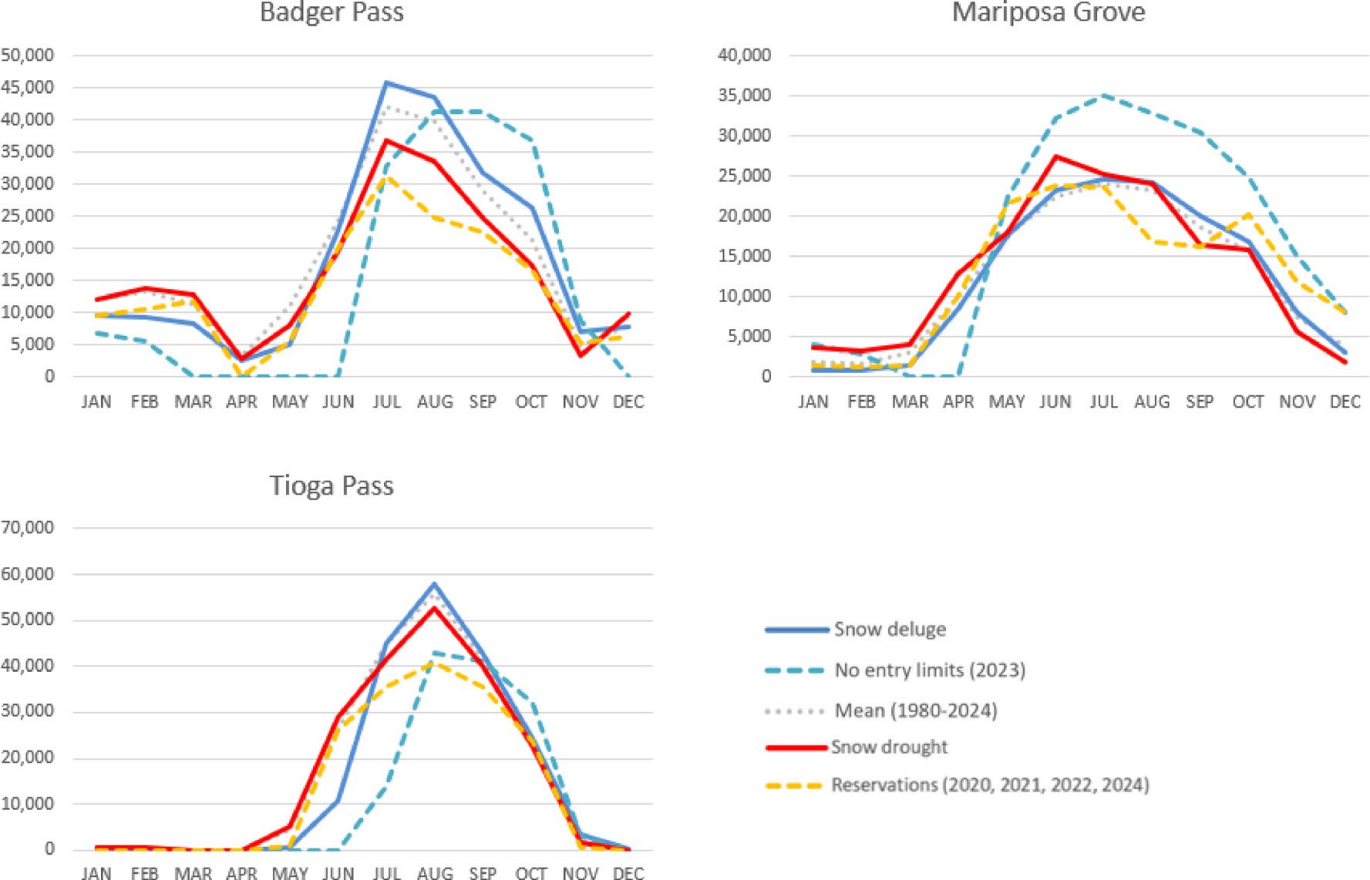
Average monthly vehicle counts at Yosemite NP (1985–2024) by climate conditions and managed access status for locations with seasonal access limitations.

Vehicle access to the park was limited in early 2023 with winter storm closures and in spring due to road failure, which displaced some use to peak summer season when visitation is already high, and when heavy snowpack led to extremely late openings for seasonal roads, thereby constraining where visitors could go and leading to more crowds and congestion (Figs. 10, 11). There was greater-than-average annual use at Arch Rock entrance in 2023 (+ 19.3%), which incurred earlier season use as visitors became displaced from the Big Oak Flat Rd entrance that was closed due to a 200-ft long fissure that cracked the highway apart. Use at the Hetch Hetchy entrance in 2023 was much higher on average annually (+ 90.3%) and peaked earlier in the season as users were displaced from the closure of Tioga Pass Rd (− 32.4%). The South Entrance incurred much more use overall in 2023 than on average annually (+ 35.5%), especially during peak summer as more visitors arrived expecting to be able to access the high country became displaced from the closure of higher elevation seasonal routes that were not yet open. Use increases and stays are higher-than-average annually (+ 40.2%) at the Mariposa Grove when the road to the sequoia grove is open for buses. Mariposa Grove opened May 25^th^ in 2023, and Glacier Point Rd opened July 15th, the latest opening day on record for each location. Badger Pass Ski Area wasn’t accessible by car and was never able to open during the winter of 2023, despite it being a record snowfall year, incurring less use than average annually (− 21.4%), largely due to the severity of winter storms that prevented access to higher elevation locations. In 2023 Badger Pass still incurred more use throughout the year compared with the average annual use during recent reservation years, which were all drier years with an earlier road opening but have less visitors than the annual average (-27.2%). This effectively led to a later-than-average start for access to the high country coincident with existing high levels of use during peak summer months, during a time when snow is melting and conditions are less certain and when demand is higher, generally leading to more crowded conditions.

## Conclusions

Park-wide access decreased during reservation years (2020–2022, 2024) when compared to the average of historic visitation, which supports our hypothesis that the day use reservation system is effective in reducing total annual visitation, higher levels of which are typically associated with crowding and lasting resource impacts that can diminish visitor experiences. In reservation years, visitation also shifted earlier in the year, suggesting that the reservation system may also effectively spread visitation away from isolated peak seasons. These findings comport with the stated goals of park management, which designed the reservation system to be effective at pacing the timing and volume of visitation into areas to optimize accessibility, ensure quality visitor experiences, and protect natural and cultural resources from impairment. The reservation system is effective at spreading use out earlier and later in the season, likely allowing for more quality experiences as visitors choose to self-displace. While it was beyond our study to assess the connections between access, quality experiences, and resource protections at the sub-monthly level necessary for determining the efficacy of the reservation system in dispersing users to different sites within the park, we can say that lower crowd levels at trailheads and most viewing areas are associated with reductions in overall use during reservation periods.

Our findings support the hypothesis that average monthly vehicle traffic counts during the extreme wet, no entry limits year (2023) were greater at different park entries than during moderately dry reservations years (2020–2022, 2024). Despite intermittent park closures and impacts to visitor infrastructure during spring and record late timing for the opening of Tioga Road, record extreme wet conditions in 2023 drove higher-than-average use through the remainder of the year. This suggests that it was not just displaced spring and winter users returning during late summer and fall, to make up for their foregone recreation opportunities. Rather, the park saw more overall visitors, as users took advantage of a longer visitation window and sought out activities and destinations either directly or indirectly dependent on water resources. For seasonally accessible locations, visitation levels showed an increase in users during 2023 at the Mariposa Grove of giant sequoias. Typically, the road to the Grove, which provides bus transit, is closed due to snow through the late spring, with winter users traversing a two-mile long trail to reach the Grove in the winter. The parking lot is usually accessible through the winter, though it remained closed through the late spring in 2023. Despite the winter access restrictions, the record extreme wet conditions of 2023 had much higher levels of use during the peak season, and extended the accessible season through the end of the calendar year, which as with other destinations in the park later that year includes additional users, likely in addition to some users who were displaced from winter and late spring.

For overnight use types, backcountry camping was responsive to snow conditions, since it is not subject to the park-wide day use reservation limits because overnight use in the Yosemite Wilderness has its own reservation system, and this has facilitated record levels of use during reservation years that have also been moderately dry years^9,46^. The length of the backcountry season is extended in years of drier-than-average snow drought, and while drier conditions are expected to become more common, the expected timing and magnitude of precipitation, snowpack, and runoff has become more uncertain^15^. Conversely, during years of snow deluge, backcountry campers may not be able to access high country locations until late summer. The extreme wet conditions in 2023 displaced would-be overnight wilderness users into late summer and extended the peak season through late fall, during which those users who were displaced and additional visitors contributed to greater-than-average overnight wilderness use. The influence of these categories may be attributable to the nature of demand for permits within the structure of the reservation system, given that the availability of overnight backcountry permits greatly depends on whether Tioga Pass is open or closed, the timing of which can vary greatly from year-to-year.

Our analysis is subject to some uncertainties and limitations. The limited period of record, with only four years of reservations, somewhat limits our ability to quantitatively disentangle reservation system impacts from hydroclimate. As more years of data with reservation systems in place become available, future work could leverage these. Alternatively, syntheses using data from other highly-visited areas with reservation systems could strengthen statistical confidence in the findings. Future work could also investigate the extent to which our findings are unique to Yosemite National Park or applicable across broader geographies.

We found that annual use levels were more influenced by managed access than by climate-extremes, however, climate extremes create more variability and uncertainty. It remains to be seen how a wet year with a reservation system in place might limit the ability of visitors to self-displace their visits to spring. Overcrowding and traffic congestion in the valley during high snowpack years may be an issue, even under day use reservations, if visitors do not have places where they can disperse. High levels of use during snow drought years with the prospect of fires can displace users from one entrance to another entrance, as can happen with extreme wet conditions associated with winter storms and flooding. Reservation systems can be adaptable in responding to this climate variability and uncertainty by moderating visitation during wet years that cause extended seasonal closures by allocating fewer reservations during peak season, and in dry years, when there are more areas for visitors to disperse, via loosening restrictions and increasing reservation allocations. A flexible approach would enable the park to mitigate overcrowding and safety concerns during wet years or when other stochastic processes like wildfires impact or constrain spatial distribution of visitors. Considerations, such as impacts to high-priority park resources, and economic factors should also be incorporated into decision-making for flexible reservation systems. For instance, in contrast to the preceding years since 2020 when reservations were in place, the uptick in visitors in 2023 associated with no entry limits led to more social or visitor created trails throughout the popular meadow systems of Yosemite Valley, and the extent of these lasting impacts to soil and vegetation from compaction were further exacerbated by wetter-than-average conditions^59^. The potential for such impacts to park resources must be carefully balanced with gateway community economies that depend heavily on park visitation, and closures in peak season and off-peak season have different implications for these economies^60^. Understanding interactions between hydroclimate impacts and reservation systems across different time scales (from month-to-month, over the course of a season, and across successive years, with potential for contemporaneous and lagging impacts) is therefore essential to planning for the economic sustainability of these communities. Future research should assess snowpack volume and timing trends, and potential impacts for no entry limits happening in a drought year, much like in 2016, when Yosemite experienced record visitation of over 5-million users, driven by the Centennial of the National Park Service, but likely exacerbated by prolonged multi-year drought conditions throughout the region.

## Supplementary Information

Below is the link to the electronic supplementary material.

Supplementary Material 1

## Data Availability

The National Park Service Visitor Use Statistics used for the analysis in the study are available at the NPS Integrated Resources Management Applications (IRMA) portal via https://irma.nps.gov/Stats/. The Snow Water Equivalent data used for the analysis in the study are available from California Department of Water Resources at the California Data Exchange Center via https://cdec.water.ca.gov/snow.html.
