# Supplementary figures and images for: Snow droughts, deluge, and reservation systems interact to drive recreation access at Yosemite National Park
Source: Sci Rep. 2025 Oct 23;15:37131. doi: 10.1038/s41598-025-21022-5 (PMC12549969; doi:10.1038/s41598-025-21022-5)

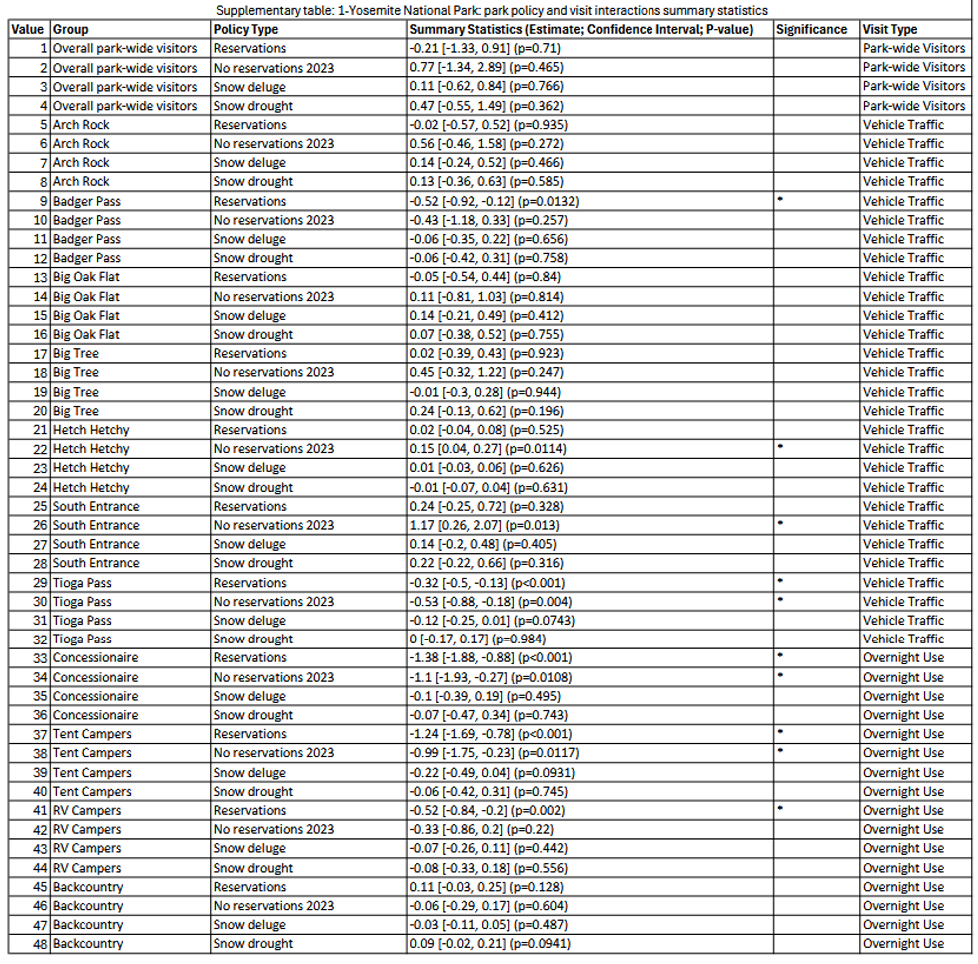

Supplement: Supplementary file 1 — Supplementary Material 1 [file 41598_2025_21022_MOESM1_ESM.png]
